# Savory biscuits formulated with mixed green banana pulp and peel flours: A sustainable approach to enhance nutritional, technological and sensory properties

**DOI:** 10.1002/jsfa.70573

**Published:** 2026-03-12

**Authors:** Leonara Martins Viana, Carlos Wanderlei Piler Carvalho, Hércia Stampini Duarte Martino, Maria Teresa Galvão, Mária Herminia Ferrari Felisberto, Frederico Augusto Ribeiro de Barros

**Affiliations:** ^1^ Department of Food Technology Federal University of Viçosa Viçosa Brazil; ^2^ Embrapa Food Technology Rio de Janeiro Brazil; ^3^ Department of Nutrition and Health Federal University of Viçosa Viçosa Brazil; ^4^ Sensenova Development and Sensory Research Barueru Brazil

**Keywords:** CATA test, consumer acceptability, fiber‐enriched savory biscuits, JAR evaluation, micro‐CT, unconventional flour

## Abstract

**BACKGROUND:**

There is a growing consumer interest in foods that not only provide nutritional value and convenience, but also promote health benefits through regular consumption. In this context, green banana flour has emerged as a promising alternative for innovative product development. This study evaluated the physicochemical, technological and sensory properties of savory biscuits formulated with green banana pulp (PF) and pulp/peel mixed flours. Four formulations were developed: a control (0‐PF), prepared with sweet and sour cassava starches, and three formulations in which 50% of the total cassava starch was replaced with green banana flours containing different pulp‐to‐peel ratios, 80‐PF (80:20), 90‐PF (90:10) and 100‐PF (100% pulp flour).

**RESULTS:**

The 80‐PF and 90‐PF formulations exhibited higher protein, lipid, and ash contents, whereas all three enriched formulations (80‐PF, 90‐PF and 100‐PF) showed increased levels of total dietary fiber and resistant starch. Texture and thickness values decreased with the increasing proportion of PF and mixed flours. Structural analysis revealed a reduction in both total and open porosity in the 80‐PF, 90‐PF and 100‐PF biscuits, which was associated with increased firmness. All formulations achieved mean sensory scores above the acceptability threshold. Notably, the 90‐PF formulation received the highest score for texture, likely due to its denser internal matrix and optimal crunchiness, as confirmed by the JAR analysis.

**CONCLUSION:**

These findings support the use of green banana pulp and pulp/peel mixed flours as functional ingredients for developing nutritionally enhanced, fiber‐rich savory biscuits with desirable textural and sensory properties. © 2026 The Author(s). *Journal of the Science of Food and Agriculture* published by John Wiley & Sons Ltd on behalf of Society of Chemical Industry.

## INTRODUCTION

Bakery products represent a promising matrix for the incorporation of functional ingredients, given their widespread consumption, generally affordable cost, long shelf life at room temperature, and substantial contribution to daily intake of energy, carbohydrates, proteins and minerals.^1^, ^2^ Although bakery products made with refined flours demonstrate favorable sensory and technological properties, they are deficient in dietary fiber and antioxidants, which limits their functional potential.^3^, ^4^, ^5^ Savory biscuits have been identified as promising vehicles for the delivery of micronutrients and bioactive compounds because they are widely consumed across all age groups and offer practicality for daily intake within busy routines.^6^

A promising strategy to enhance the nutritional quality of biscuits involves the incorporation of unconventional flours rich in bioactive compounds, such as those derived from unripe bananas. These flours are predominantly produced from the pulp, which is notably rich in resistant starch (RS), a compound associated with several health benefits, including glycemic control and modulation of gut microbiota.^7^, ^8^, ^9^, ^10^, ^11^ As a result of their composition, green banana pulp flours can be successfully incorporated into a wide range of cereal‐based products, including bread, extrudates, cookies, snacks, crackers and pasta.^12^, ^13^, ^14^, ^15^, ^16^ Although their addition can significantly enhance the nutritional profile of these products, it may also impact technological and sensory attributes, such as texture, color, and processing variables, which should be carefully considered during formulation.

In addition to the pulp, green banana peel flour has attracted growing interest due to its notable nutritional and functional properties. As a by‐product of banana processing, it contains higher levels of dietary fiber, minerals and antioxidants compared to the green banana pulp flour.^17^ Its use also presents an environmentally sustainable strategy by reducing food waste and adding value to agro‐industrial residues. In this context, several studies have reported positive effects of incorporating banana peel flour into baked goods. Bakare *et al*.^18^ demonstrated that the addition of up to 20% green banana peel flour preserved the textural and sensory properties of the product while significantly increasing the concentration of dietary fiber and total phenolic compounds. Similarly, Mahloko *et al*.^19^ observed that the inclusion of banana peel flour, either alone or in combination with prickly pear peel flour, enhanced the phenolic and fiber content, and improved antioxidant activity, without negatively affecting the textural profile of the biscuits.

Although green banana pulp and peel flours have been studied separately in bakery products, their combined use in savory biscuits has not been explored. The diversity of the nutritional profile in pulp and peel flours, such as resistant starch, dietary fiber, minerals and antioxidants, suggests that their combination could enhance both the functional and technological properties of the final product. Moreover, the use of both fractions enables the integral utilization of green bananas, supports sustainable food production by valorizing agro‐industrial by‐products, and allows for potentially gluten‐free formulations. This approach represents a novel strategy for developing biscuits with enhanced nutritional value, potential health benefits and environmental sustainability. Therefore, the present study aimed to develop savory biscuits enriched with mixed green banana pulp and peel flours in ratios of 90:10 and 80:20 (pulp: peel) and to evaluate their physicochemical, technological and sensory characteristics, highlighting the novel potential of this formulation for functional and sustainable bakery products.

## MATERIALS AND METHODS

### Preparation of green banana pulp and peel flours

Green banana (*Musa* spp.) bunches (cultivar ‘Prata’, genome group AAB) were obtained in Coimbra, Minas Gerais, Brazil, and processed into green banana pulp flour (PF) and peel flour (PeF). Briefly, the fruits were washed, sanitized, manually peeled, and separated into pulp and peel, which were subsequently sliced and treated to prevent enzymatic browning. The materials were then dried at 55°C for 9 h in a gas‐heated tray dryer, with the airflow velocity adjusted to 1.5 m s^−1^, and milled to obtain the respective flours, following a previously established method developed by our research group.^17^ The mixed flours were prepared by combining 90% PF and 10% PeF (MPF90) and 80% PF and 20% PeF (MPF80) using manual mixing for 1 min in a plastic container.^17^ Additional ingredients, including milk, sweet cassava (*Manihot esculenta* Crantz) starch and sour cassava (*Manihot esculenta* Crantz) starch, salt, eggs and unsalted margarine were purchased from a local supermarket in Viçosa, Minas Gerais, Brazil.

### Characterization of green banana pulp and peel flours

#### Proximate composition

The moisture, protein, lipid and ash contents of the flours were determined by the following AACC methods: 44–15.02, 46–13.01, 30–25.01 and 08–01.01,^20^ respectively. Soluble and insoluble fibers were quantified by the enzymatic‐gravimetric method, using thermo‐resistant α‐amylase, protease, and amyloglucosidase (Megazyme; Neogen, Lansing, MI, USA) for enzymatic hydrolysis.^21^

Total dietary fiber was determined according to AACC method 32‐07.01.^20^ The digestible carbohydrate content was calculated by difference. The resistant starch (RS) content was determined according to the official AACC method 32‐40.01 AACC^22^ using a commercial assay kit from Megazyme International (Wicklow, Ireland).

#### Mineral content

The determination of the mineral composition [phosphorus (P), calcium (Ca), magnesium (Mg), sulfur (S), boron (B), zinc (Zn), copper (Cu), manganese (Mn), iron (Fe) and sodium (Na)] of the green banana pulp and peel flours was determined as proposed by Oliveira *et al*.^23^

### Formulation of the savory biscuits

The biscuit formulations were developed by ourselves based on preliminary formulation trials aimed at optimizing the nutritional and sensory quality of the final product. The proportions of ingredients used in the formulations are presented in Table 1. A control sample of savory biscuits prepared with a blend of sweet cassava starch and sour cassava starch was produced, along with three additional formulations in which 50% of this starch blend was replaced by green banana pulp flour (PF) or by mixed pulp/peel flours. Thus, four formulations were obtained (Table 1): 0‐PF (control, 0% PF), 100‐PF (100% PF), 90‐PF (MPF90) and 80‐PF (MPF80).

**Table 1:** Base formulation for preparation of the savory biscuits

Ingredients (g per 100 g)	0‐PF	80‐PF	90‐PF	100‐PF
Unsalted margarine	20	20	20	20
Egg yolk	14	14	14	14
Lemon pepper	2	2	2	2
Salt	1	1	1	1
Water	60	60	60	60
Flour‐based ingredients^a^				
Pulp flour (PF)	0	40	45	50
Peel flour (PeF)	0	10	5	0
Sweet cassava starch	50	25	25	25
Sour cassava starch	50	25	25	25

^a^ The flour‐based ingredients correspond to 100 g of the flour fraction. 0‐PF (control, 0% PF); 80–PF (formulation elaborated with MPF80); 90–PF (formulation elaborated with MPF90); and 100–PF (formulation elaborated with 100% PF).

For biscuit preparation, unsalted margarine, salt and water were weighed according to each formulation (g per 100 g of dough) (Table 1), manually mixed and heated on a conventional household stove (Dako, Rio de Janeiro, Brazil) until boiling, reaching 87 °C as monitored using a digital culinary thermometer (approximately 5 min). The blend of sweet and sour cassava starches and lemon pepper were then added to the heated mixture and manually mixed for 2 min. Egg yolks and PF or the mixed pulp/peel flours (MPF90 and MPF80) were gradually incorporated until a homogeneous dough was obtained. The resulting dough was laminated using a pasta roller attachment (KitchenAid, Cassarà, Italy) at 39 mm thickness, cut into rectangular shapes (9 cm in length) and baked in a household oven (Fischer, São Paulo, Brazil) at 180 ± 4 °C for 12 ± 1 min. For each formulation, two batches were prepared, and after cooling, the biscuits were packaged and stored at −20 °C.

### Physicochemical properties of the savory biscuits

No analyses were conducted at the dough stage; all product‐related measurements were performed on the baked biscuits. For chemical analyses, the biscuits were packaged and stored at −20 °C until analysis. Except for texture, which was evaluated 24 h after baking, the other techno‐functional analyses, including physical, color and structural measurements, as well as sensory evaluation, were performed on baked biscuits after cooling to room temperature.

The moisture, protein, lipid, ash and carbohydrate contents, as well as soluble and insoluble fiber fractions, total dietary fiber and resistant starch content (RS), were determined using the same equipment and parameters as described in the section above on Proximate composition.

### Technological properties of savory biscuits

The specific volume was calculated as the ratio of apparent volume to weight. Apparent volume (mL) was measured by seed displacement, according to AACC method 10‐05.01^20^ and weight (g) was determined using a Shimadzu UX‐6200H semi‐analytical balance (Shimadzu Corporation, Kyoto, Japan). Biscuit diameter was evaluated by using a Vernier calliper at five different places of 10 randomly selected samples. The weight loss was calculated as a ratio between the pre‐and post‐cooking by products.

Water activity (*a*_w_) was measured directly in a 4TE Aqualab apparatus (Decagon Devices Inc., Pullman, WA, USA), at a controlled temperature of 25.30 °C.

Instrumental color analysis was performed on biscuits using a Minolta Color Reader CR‐10 (Konica Minolta Co., Aichi, Japan), with a 50‐mm port size, illuminant D65, specular component included and a 10° standard observer angle. The color of the biscuits was evaluated using the tri‐stimulus CIELab color space method, determining the lightness (L*), redness (a*) and yellowness (b*) values. The whiteness index (WI) and browning index (BI) were determined according to the method proposed by Gat & Ananthanarayan.^24^ The total color difference (ΔE*) was calculated following the procedure described by Laguna *et al*.^25^

Additionally, biscuit hardness was evaluated using a TA‐XT Plus texture analyzer (Stable Micro Systems, Godalming, UK) equipped with a tree‐point bending rig. The test was performed with a trigger force of 25 g and a 50 kg load cell. Pre‐test, test and post‐test speeds were set at 1.5, 2.0 and 10.0 mm s^−1^, respectively, following the method described by Jan *et al*.^26^ The compression distance was 10 mm, and the distance between the two bottom supports was 4 mm. All measurements were performed in quintuplicate, with two independent repetitions.

The structure characteristics of the biscuit samples were non‐destructively investigated using an 3D X‐ray micro‐computed tomography (Micro‐CT) system (SkyScan 1174; Bruker, Kontich, Belgium). The power settings used were 800 μA/40 kV, with a pixel size of 29.90 μm for the samples. Reconstruction was performed with NRecon software version 1.7.0.4 (Bruker, Konitich, Belgium) with smoothing at 06, ring artifact correction at 15 and beam hardening at 20%. Tomographic parameters were measured in triplicate at the exact center of each savory biscuit, using a 4‐mm thick cross‐sectional slice.

The percentage of total material volume (ObjV1/TV, in %); total volume of pore space (Po.V, in mm^3^), as well as the percentage of the closed, open and total porosity, and the number of closed pores (cm^3^) were then analyzed using CT Analyser, version 1.23.0.2 (Bruker) and 3D models were created with CTVox software, version 3.2.0 (Bruker).

### Sensory evaluation of savory biscuits

Following approval from the Ethics Committee on Human Research of the Federal University of Vicosa (Protocol number: 6.509.538; CAAE: 71589423.0.000.5153), 120 untrained panelists (males and females, aged 18–60 years) without banana allergies were recruited and screened. The participants included students and employees from the campus. Prior to the sensory evaluation, the research objectives were properly communicated, and all subjects provided their written informed consent.

On the day of analysis, samples from each treatment were baked in batches of four loaves per session. Specifically, aluminum loaf pans containing the different treatments were baked simultaneously in the same oven under identical conditions, and this process was repeated for each session conducted. Each participant evaluated four samples per session, and each savory biscuit provided to the panelists had a standardized average weight of approximately 1.20 g and an average length of 0.60 cm. Samples were monadically served in disposable plates coded with three‐digit random numbers, following a balanced and randomized presentation order according to a randomized complete block design, in which all panelists evaluated all samples, and the serving order was randomized across participants to minimize order and carry‐over effects. Panelists rinsed their mouths with drinking water at room temperature between samples to minimize residual flavors.

#### Acceptance test and purchase intent

Savory biscuits samples were submitted to the acceptance test using a nine‐point structured hedonic scale ranging from ‘disliked extremely’ (1) to ‘liked extremely’ (9) for appearance, flavor, aroma, texture and overall liking.^27^ Purchase intent was measured on a five‐point scale, ranging from ‘1’ (certainly would not buy) to ‘5’ (certainly would buy).^28^

#### Check‐all‐that‐apply (CATA) and just‐about‐right (JAR)

Consumers were instructed to select all the terms they considered applicable to describe each sample using the CATA question format.^29^ This question consisted of a list of 18 terms, including both descriptive and hedonic attributes, from which they should check all the attributes they considered that applied to describe each biscuit formulation. The list of terms was compiled using an online questionnaire and based on the study by Di Cairano *et al*.,^2^ with modifications. The order of attributes of each category was balanced among consumers.^29^ Additionally, the biscuit samples were evaluated for crunchiness, color, spices, and saltiness intensity using a five‐point JAR scale, anchored at ‘1’ for much less and ‘5’ for much more, with the central point ‘3’ for ideal, to assess the perceived levels of these attributes in the products.^30^

### Statistical analysis

For the characterization of the flours, mean comparisons were performed using Student's *t*‐test. The physicochemical and technological properties of the biscuit formulations were analyzed by one‐way analysis of variance (ANOVA). When significant differences were detected (*P* < 0.05), treatment means were compared using Tukey's post‐hoc test. Statistical analyses were performed using Prism, version 8.0 (GraphPad Software Inc., San Diego, CA, USA) and the results are presented as the mean ± SD.

Regarding sensory analyses, consumer liking scores were analyzed by ANOVA, considering sample (formulation) as the main effect. When significant differences were detected (*P* < 0.05), treatment means were compared using Tukey's post‐hoc test. CATA data were first evaluated to determine whether consumers detected differences between samples based on the proposed attributes using Cochran's *Q* test, followed by multiple pairwise comparisons with the critical difference (Sheskin) procedure. Penalty lift analysis was performed to assess the impact of each attribute on the liking score (*P* < 0.05). The five‐point JAR scale was categorized into three levels: much less, JAR, and much more, then, penalty (or mean drop) analysis was conducted.^30^ All sensory data analyses were performed using XLSTAT 2023.3 (Addinsoft, New York, NY, USA), considering a significance level of *P* < 0.05 for all tests.

## RESULTS AND DISCUSSION

### Proximate composition and mineral content of green banana pulp and peel flours

The proximate composition of green banana pulp flour (PF) and peel flour (PeF) is presented in Table 2. PF exhibited a notably higher content of resistant starch (42.58 g per 100 g), whereas PeF showed significantly (*P* < 0.05) higher levels of ash, proteins and lipids, corroborating our previous findings.^17^ Regarding dietary fiber, the insoluble fraction (36.85 g per 100 g) comprised the majority of the composition of PeF, whereas PF contained considerably lower fiber content, with insoluble fiber accounting for only 5.19 g per 100 g. Moreover, PeF exhibited a substantially higher mineral content than PF, being particularly rich in potassium, calcium, phosphorus, magnesium and iron, consistent with our previous findings.^17^

**Table 2:** Proximate composition of the green banana pulp and peel flours

Components (g per 100 g)^a^	PF	PeF
Moisture	4.07 ± 0.60 b	7.01 ± 0.29 a
Lipids	0.35 ± 0.03 b	7.41 ± 0.04 a
Protein	4.37 ± 0.34 b	6.61 ± 0.00 a
Ash	2.02 ± 0.02 b	9.61 ± 0.27 a
Total carbohydrate	89.19 ± 0.18 a	69.36 ± 0.09 b
Resistant starch	42.58 ± 3.67 a	21.00 ± 0.87 b
Dietary fiber		
TDF	7.07 ± 0.23 b	39.57 ± 1.23 a
SDF	1.89 ± 0.17 a	3.62 ± 0.51 a
IDF	5.19 ± 0.05 b	36.85 ± 0.71 a
Minerals		
Potassium	1.014 ± 0.004 b	2.554 ± 0.175 a
Calcium	0.0176 ± 0.0015 b	0.310 ± 0.003 a
Phosphorus	0.101 ± 0.001 b	0.1897 ± 0.0015 a
Magnesium	0.131 ± 0.0021 b	0.2416 ± 0.0012 a
Sulfur	0.030 ± 0.001 b	0.0576 ± 0.0006 a
Sodium	0.002 ± 0.000 b	0.00367 ± 0.0006 a
Copper	0.0005 ± 0.00009 a	0.00058 ± 0.0001 a
Iron	0.0009 ± 0.0001 b	0.00426 ± 0.0001 a
Zinc	0.0011 ± 0.0001 b	0.00476 ± 0.0002 a
Manganese	0.0067 ± 0.00007 b	0.0214 ± 0.0002 a
Boron	0.0005 ± 0.00001 b	0.00157 ± 0.00006 a

Results are expressed as the mean ± SD. Means followed by the same lowercase letter in a row (PF *vs*. PeF) do not differ by Student's *t*‐test (*α* = 0.05).

^a^ Dry basis.

Abbreviations: PF, green banana pulp flour; PeF, green banana peel flour; TDF, total dietary fiber; SDS, soluble dietary fiber; IDF, insoluble dietary fiber.

The differences in the chemical composition of PF and PeF, particularly in protein, ash, lipids and dietary fiber, underscore the nutritional complementarity of the two flours and support their combined use to simulate a ‘whole green banana flour’ (i.e., incorporating both pulp and peel). Considering that the peel accounts for approximately 40% of the total fruit weight,^31^ the estimated composition of such whole flour would be 5.31 g per 100 g of protein, 4.86 g per 100 g of ash, 3.17 g per 100 g of lipids and 22.17 g per 100 g of total dietary fiber, predominantly insoluble fiber (19.59 g per 100 g). These results highlight the potential of combining PF and PeF to enhance the nutritional profile of baked products.

### Physicochemical properties of the savory biscuits

The proximate composition of the savory biscuit samples is presented in Table 3. Increasing the level of PeF in the formulations resulted in a significant (*P* < 0.05) decrease in moisture content and an increase in lipid content. Protein and ash contents also rose significantly (*P* < 0.05) with higher PeF incorporation, which was expected given the higher protein (6.61%) and ash (9.61%) levels in PeF compared to PF (4.37 and 2.02%, respectively). Considering these changes, along with the mineral profile of PeF, the enriched formulations are expected to contain higher amounts of essential minerals such as potassium and magnesium, thereby enhancing their nutritional value. Total carbohydrate content ranged from 71.90% to 77.04%, with the control sample (0‐PF) showing the highest value, reflecting its lower protein, lipid and ash contents compared to the enriched formulations.

**Table 3:** Proximate composition of the baked savory biscuits formulated with green banana pulp flour and/or mixed pulp/peel

Components (per 100 g)^a^	Formulations			
	0‐PF	80‐PF	90‐PF	100‐PF
Moisture	5.36 ± 0.24 b	3.80 ± 0.04 c	5.67 ± 0.17 b	6.29 ± 0.10 a
Lipids	15.97 ± 0.03 b	17.57 ± 0.06 a	16.34 ± 0.39 a	15.34 ± 0.17 b
Protein	0.82 ± 0.58 b	3.30 ± 0.01 a	4.11 ± 0.02 a	2.89 ± 0.47 a
Ash	1.77 ± 0.03 d	3.43 ± 0.18 a	2.65 ± 0.35 bc	2.37 ± 0.39 cd
Total carbohydrate	77.04 ± 0.16 a	71.90 ± 0.89 bc	72.70 ± 1.27 bc	73.93 ± 0.10 ab
Resistant starch	0.84 ± 0.10 c	5.09 ± 0.60 b	5.28 ± 0.50 b	7.42 ± 0.46 a
Dietary fiber				
TDF	1.03 ± 0.32 c	7.34 ± 0.25 a	6.18 ± 0.69 a	4.24 ± 0.22 b
SDF	0.22 ± 0.06 b	0.53 ± 0.05 a	0.72 ± 0.07 a	0.61 ± 0.11 a
IDF	0.81 ± 0.40 c	6.84 ± 0.29 a	5.45 ± 0.62 a	3.63 ± 0.11 b

0‐PF (control, 0% PF); 80‐PF (formulation elaborated with MPF80); 90‐PF (formulation elaborated with MPF90) and 100‐PF (formulation elaborated with 100% PF). TDF: total dietary fiber; SDS: soluble dietary fiber; IDF: insoluble dietary fiber. Results are expressed as the mean ± SD. Means followed by the same lowercase letter in a row do not differ at 5% probability by the *F*‐test (ANOVA).

^a^ Dry basis.

As expected, biscuits made with PF or pulp/peel mixed flours (MPF90 and MPF80) exhibited higher RS levels than the control formulation (0‐PF) (Table 3). In the raw dough, RS content varied according to the proportion of green banana flours used, reaching 10.81 g per 100 g in the 100‐PF formulation, 10.25 g per 100 g in 90‐PF and 9.71 g per 100 g in 80‐PF. After baking, the 80‐PF and 90‐PF formulations retained more than 5 g per 100 g of RS, whereas 100‐PF recorded the highest post‐baking value (7.42 g per 100 g). These correspond to RS retention rates of 60.87, 52.29, and 49.73%, respectively. This retention is attributed to the granular starch structure, which can be preserved in low‐moisture products such as biscuits.^32^ The high RS retention is also associated with the dry‐heat processing method employed.^33^ Consistent with these results, Queiroz *et al*.^32^ reported that dry‐heat treatment allowed RS retention levels ranging from 49.8 to 92.7% in cookies compared to their respective doughs.

Additionally, the 80‐PF and 90‐PF formulations exhibited the highest total dietary fiber (TDF) contents, followed by 100‐PF (7.34%, 6.18% and 4.24%, respectively), whereas the control sample (0‐PF) showed the lowest value (1.03%), as expected (Table 3). This pattern was consistent for both soluble (SDF) and insoluble dietary fiber (IDF), which increased notably in the 80‐PF, 90‐PF and 100‐PF formulations as a result of the incorporation of green banana pulp and mixed pulp/peel flours. Given the functional role of dietary fiber, such increases may be nutritionally relevant because fiber supplementation has been associated with reduced eating frequency and food intake, potentially contributing to weight loss.^34^

In light of these results and considering the World Health Organization's recommended dietary fiber intake for healthy adults (25–30 g per day), a 50 g portion of the control formulation (0‐PF) would provide only 1.35% of the daily requirement. By contrast, the same portion (equivalent to a typical snack package) of formulations enriched with green banana pulp or mixed pulp/peel flours would supply more than 5% of the recommendation. Thus, even a small serving of these enriched savory biscuits could meaningfully contribute to daily fiber intake and support healthier eating habits, particularly for individuals with busy lifestyles.

### Technological properties of savory biscuits

The physical properties of the biscuits, including weight loss, specific volume, thickness, height, water activity, color, and hardness, are presented in Table 4. Weight loss varied significantly among formulations (*P* < 0.05), ranging from 28.34% to 30.33%. This finding is consistent with the moisture content results (Table 3) because greater weight loss is associated with lower moisture levels. The highest values were observed in biscuits made with mixed pulp/peel flours, whereas the lowest were found in the 100‐PF and control (0‐PF) samples (28.34% and 28.59%, respectively), both lacking PeF. Although the incorporation of mixed flours significantly affected weight loss, the addition of PF alone did not produce a notable effect. This is likely a result of the high insoluble fiber content of PeF, which promotes water evaporation during baking.^17^

**Table 4:** Physical properties of savory biscuit baked

	Formulations			
	0‐PF	80‐PF	90‐PF	100‐PF
Weight loss (%)	28.34 ± 0.18 c	29.75 ± 2.63 b	30.33 ± 0.69 a	28.59 ± 0.34 c
Specific volume (cm^3^ g^−1^)	3.74 ± 0.07 a	4.85 ± 2.23 a	4.25 ± 0.13 a	5.01 ± 3.18 a
Thickness (cm)	0.57 ± 0.03 a	0.33 ± 0.02 bc	0.31 ± 0.02 c	0.27 ± 0.02 dc
Height (cm)	0.63 ± 0.06 a	0.61 ± 0.04 a	0.62 ± 0.00 a	0.61 ± 0.01 a
Water activity	0.27 ± 0.005 cd	0.37 ± 0.005 a	0.26 ± 0.002 d	0.28 ± 0.008 bc
Color parameters				
L*	71.00 ± 0.36 a	50.32 ± 0.73 d	53.29 ± 0.28 c	57.54 ± 1.24 b
a*	6.68 ± 0.25 c	7.31 ± 0.18 b	9.00 ± 0.17 a	8.86 ± 0.12 a
b*	27.02 ± 0.25 a	18.62 ± 0.50 c	21.47 ± 0.12 b	22.18 ± 0.33 b
Δ*E*	–	22.33	18.70	14.47
WI	59.80 ± 0.19 a	46.44 ± 0.53 c	47.81 ± 0.26 c	51.28 ± 0.94 b
BI	53.38 ± 0.23 d	55.60 ± 1.13 c	62.46 ± 0.72 a	58.62 ± 0.86 b
Hardness (N)	6.12 ± 1.71 a	2.19 ± 0.43 b	1.89 ± 0.36 b	0.72 ± 0.11 c

0‐PF (control, 0% PF); 80‐PF (formulation elaborated with MPF80); 90‐PF (formulation elaborated with MPF90) and 100‐PF (formulation elaborated with 100% PF). ΔE: numerical total color difference; WI: whiteness index; BI: browning index. Results are expressed as the mean ± SD. Means followed by the same lowercase letter in a column do not differ at 5% probability by Tukey's test.

Formulations enriched with green banana pulp or mixed pulp/peel flours showed no significant differences in specific volume or height compared with the control biscuit. However, the control (0‐PF) exhibited significantly greater thickness (*P* < 0.05) than the 80‐PF, 90‐PF and 100‐PF formulations. This reduction in thickness in the enriched samples may be attributed to their higher fiber content relative to the control.^35^ All savory biscuit formulations had low water activity values (0.26–0.37), which contribute to greater chemical and microbiological stability and enhanced crispness.^14^

The addition of PF, MPF90 or MPF80 flours to the biscuit formulations resulted in lower luminosity (L) and whiteness index (WI) values, along with higher browning index (BI) values. This was expected, as green banana pulp flour has a slightly beige to yellowish color,^17^ which darkens the product compared with the control. This difference was confirmed by the total color difference (Δ*E*), with the most pronounced changes observed in the 80‐PF and 90‐PF formulations (Table 4). These variations are also evident as shown in the Supporting information (Fig. S1), which visually illustrates the color differences among formulations. Changes in *a* and *b* values further highlight these variations: while the control biscuit (0‐PF) displayed a reddish hue (*b*) with moderate yellowness (*a*), enriched samples exhibited slightly more intense red tones and reduced yellowness. Such color changes may also be attributed to the formation of brown pigments through the Maillard reaction.^36^

Regarding texture, significant reductions (*P* < 0.05) in hardness were observed in formulations containing green banana flour (Table 4). The control sample (0‐PF) was the hardest (6.12 N), whereas the 100‐PF formulation exhibited the lowest hardness (0.72 N), with 80‐PF and 90‐PF showing intermediate values (2.19 N and 1.89 N, respectively). This effect is likely related to the higher fiber content of these flours, which can alter dough structure by compacting the matrix.^37^, ^38^ Similar results were reported by Adeyemo *et al*.,^39^ where the inclusion of unripe plantain flour reduced biscuit hardness compared to a market sample. Bouazizi *et al*.^40^ further noted that dietary fiber‐rich ingredients interact with the water‐holding capacity of the matrix, influencing moisture distribution and contributing to a crispier or crunchier texture in baked products.

**Figure 1:**
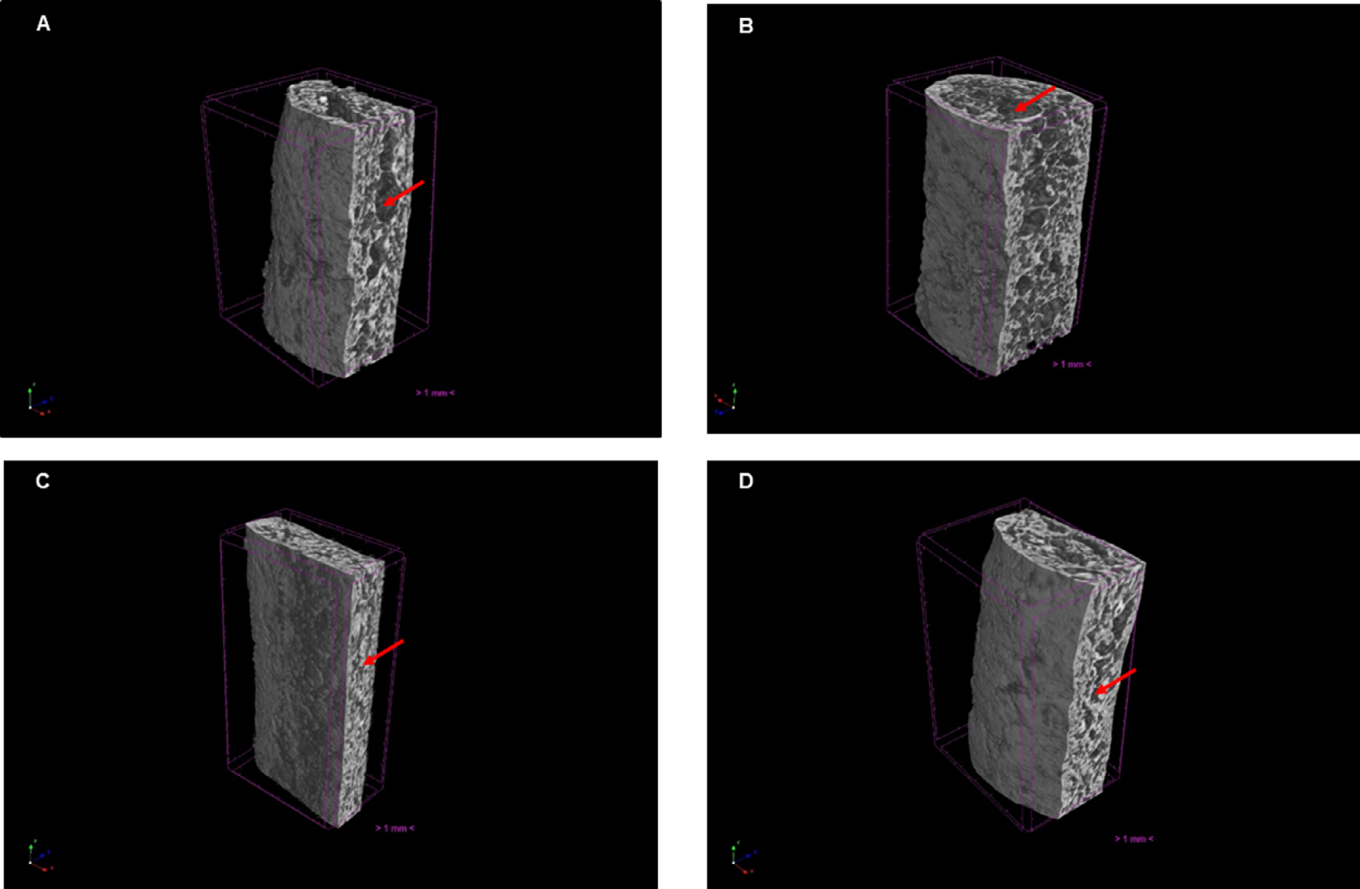
Three‐dimensional (3D) reconstructions of the internal structure of savory biscuits obtained by X‐ray microtomography, illustrating the effect of green banana pulp and/or mixed pulp/peel flours on the biscuit matrix. (A) 0‐PF (control, 0% PF); (B) 80‐PF (formulation elaborated with MPF80); (C) 90‐PF (formulation elaborated with MPF90); and (D) 100‐PF (formulation elaborated with 100% PF).

3D tomographic analysis revealed significant structural differences in biscuits enriched with PF and mixed pulp/peel flours (MPF80 and MPF90). Compared with the control (0‐PF), the 80‐PF, 90‐PF and 100‐PF formulations exhibited significantly higher ObjV1/TV values and lower total and open porosity (Fig. 1 and Table 5), indicating a denser, less porous matrix, comprising findings consistent with the increased firmness observed in the texture profile (Table 4). These changes are likely a result of the high dietary fiber content of green banana flours, which promotes dough compaction and limits matrix expansion during baking.^41^ Similar trends were reported by Khoza *et al*.,^42^ who found that the dense starch granules and high resistant starch content of green banana flour enhance structural cohesion and reduce porosity in bakery products. No significant differences were observed in the number of closed pores among the enriched formulations, suggesting that fiber contributed to a uniformly compact internal structure. In contrast, the control formulation (0‐PF), composed exclusively of starch‐rich ingredients, may have undergone more extensive starch gelatinization,^43^ leading to distinct microstructural characteristics (Table 5).

**Table 5:** Overview of the 3D morphological parameters for the three cross sections of savory biscuits

Parameters		0‐PF	80‐PF	90‐PF	100‐PF
ObjV1/TV (%)		98.40 ± 0.22 b	99.17 ± 0.07 a	99.68 ± 0.02 a	99.43 ± 0.15 a
Po.V(tot) (mm^3^)		0.84 ± 0.12 a	0.36 ± 0.03 b	0.11 ± 0.01 c	0.22 ± 0.05 c
Porosity (%)	Closed	0.53 ± 0.12 b	0.59 ± 0.06 b	1.12 ± 0.24 a	0.87 ± 0.02 a
	Open	1.08 ± 0.31 a	0.24 ± 0.13 b	0.00 ± 0.00 b	0.00 ± 0.00 b
	Total	1.60 ± 0.22 a	0.83 ± 0.07 b	0.32 ± 0.02 b	0.57 ± 0.15 b
Number of closed pores (cm^3^)		915.00 ± 40.63 a	843.00 ± 7.94 a	1078.67 ± 76.51 a	1043.67 ± 225.08 a

ObjV1/TV: total material volume; Po.V(tot): total volume of pore space; 0‐PF (control, 0% PF); 80‐PF (formulation elaborated with MPF80); 90‐PF (formulation elaborated with MPF90) and 100‐PF (formulation elaborated with 100% PF). Means followed by the same lowercase letter in a row do not differ at 5% probability by the *F*‐test (ANOVA).

### Sensory properties of savory biscuits

The overall acceptance scores for the savory biscuit formulations are presented in Table 6. The control sample (0‐PF) received the highest ratings for appearance and color (6.70 and 6.60, respectively), likely reflecting consumer familiarity with refined starch‐based products. Nevertheless, all formulations containing PF or mixed flours (MPF80 and MPF90) scored above the acceptability threshold (score ≥ 5), indicating positive sensory acceptance.

**Table 6:** Overall liking scores of savory biscuits enriched with pulp flour and mixed pulp/peel flours

Attributes^a^	Formulations			
	0‐PF	80‐PF	90‐PF	100‐PF
Appearance	6.70 ± 1.90 a	5.40 ± 2.01 b	5.60 ± 1.77 b	5.80 ± 1.76 b
Color	6.60 ± 1.96 a	5.30 ± 1.98 b	5.50 ± 1.85 b	5.70 ± 1.82 b
Aroma	6.30 ± 1.63 a	6.40 ± 1.42 a	6.40 ± 1.45 a	6.10 ± 1.65 a
Texture	5.90 ± 1.84 c	6.70 ± 1.75 b	7.30 ± 1.25 a	6.71 ± 1.85 ab
Flavor	6.50 ± 1.96 a	6.90 ± 1.57 a	7.10 ± 1.57 a	6.50 ± 1.84 a
Overall acceptance	6.40 ± 1.62 a	6.50 ± 1.46 a	6.80 ± 1.48 a	6.30 ± 1.63 a
Purchase intention	3.20 ± 1.16 ab	3.20 ± 1.07 ab	3.50 ± 1.06 a	3.10 ± 1.15 b

^a^ 9‐point liking response scale: 1 = dislike extremely, 9 = like extremely. 0‐PF (control, 0% PF); 80‐PF (formulation elaborated with MPF80); 90‐PF (formulation elaborated with MPF90) and 100‐PF (formulation elaborated with 100% PF). Results are expressed as the mean ± SD. Means followed by the same lowercase letter in a row indicate significant differences by Tukey's test (*P* < 0.05).

In terms of texture, the control (0‐PF) obtained the lowest mean score (5.90), whereas 90‐PF achieved the highest (7.30), suggesting a particularly favorable perception of texture in this formulation. This enhanced texture acceptability may be linked to greater structural cohesion, as evidenced by the lower porosity observed in 90‐PF (Table 5) and/or to the compositional diversity of the mixed flours, which may produce a denser internal matrix and more desirable mouthfeel. These findings are further supported by the higher proportion of ideal responses in the JAR evaluation of crunchiness for 90‐PF, reinforcing the connection between internal structure and sensory perception.^42^ No significant differences were observed between enriched formulations (80‐PF, 90‐PF and 100‐PF) and the control (0‐PF) in overall acceptance (6.30–6.80) or purchase intention (3.10–3.50).

**Figure 2:**
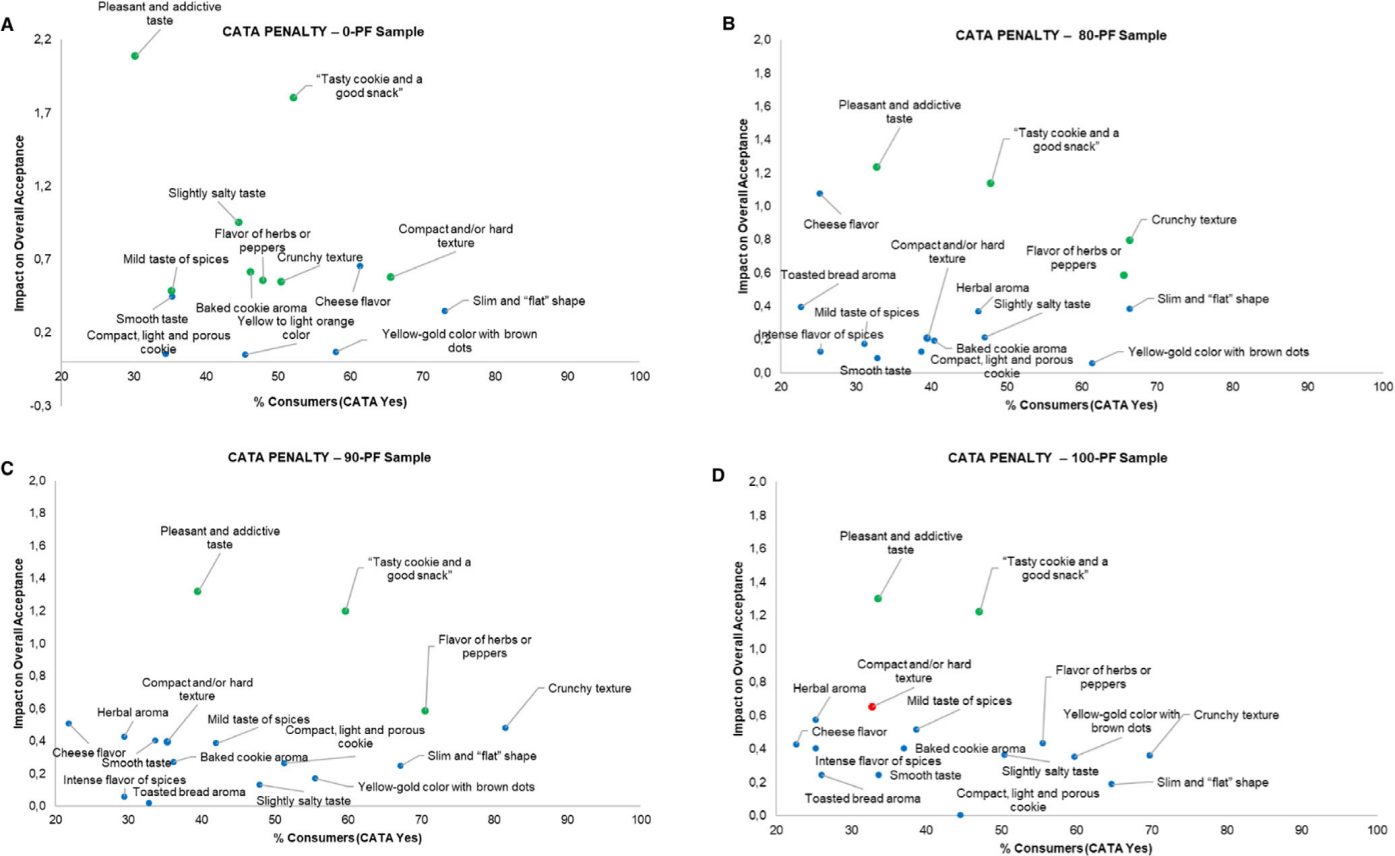
Correspondence analysis of CATA frequencies for the savory biscuit samples. (A) 0‐PF (control, 0% PF); (B) 80‐PF (formulation elaborated with MPF80); (C) 90‐PF (formulation elaborated with MPF90); and (D) 100‐PF (formulation elaborated with 100% PF). Attributes represented in green (+) and red (−) had a significant positive or negative impact on overall liking, according to Cochran's *Q* test at 95% significance level. Blue attributes showed no significant impact.

Additionally, the CATA analysis of savory biscuits formulated with green banana pulp or pulp/peel mixed flours indicates that a balanced combination of saltiness, aroma, and texture is essential for optimizing the sensory experience of consumers (Fig. 2; see also Supporting information, Table S1). Attributes such as ‘pleasant and addictive taste’, ‘tasty biscuit and a good snack’ and ‘crunchy texture’ were pivotal drivers of positive acceptance (*P* < 0.05) for the 80‐PF, 90‐PF and 100‐PF formulations. Furthermore, the flavor of ‘herbs or peppers’ emerged as a significant positive attribute in the 80‐PF formulation, suggesting that these sensory characteristics enhance the overall complexity and consumer appeal of the product.

The higher proportion of sweet and sour cassava starches in the 0‐PF formulation may have negatively contributed to the ‘compact texture’ attribute, as indicated by the CATA results (66%, *P* = 0.014). However, this attribute did not significantly impact consumer perception in the 80‐PF and 90‐PF formulations, suggesting greater acceptance of these samples by panelists. This difference could be attributed to the type and quantity of ingredients used, particularly the protein and fiber content of the flour, which are known to significantly affect physical properties such as hardness and porosity,^44^, ^45^, ^46^, ^47^, which is a trend also observed in our enriched biscuit formulations.

**Figure 3:**
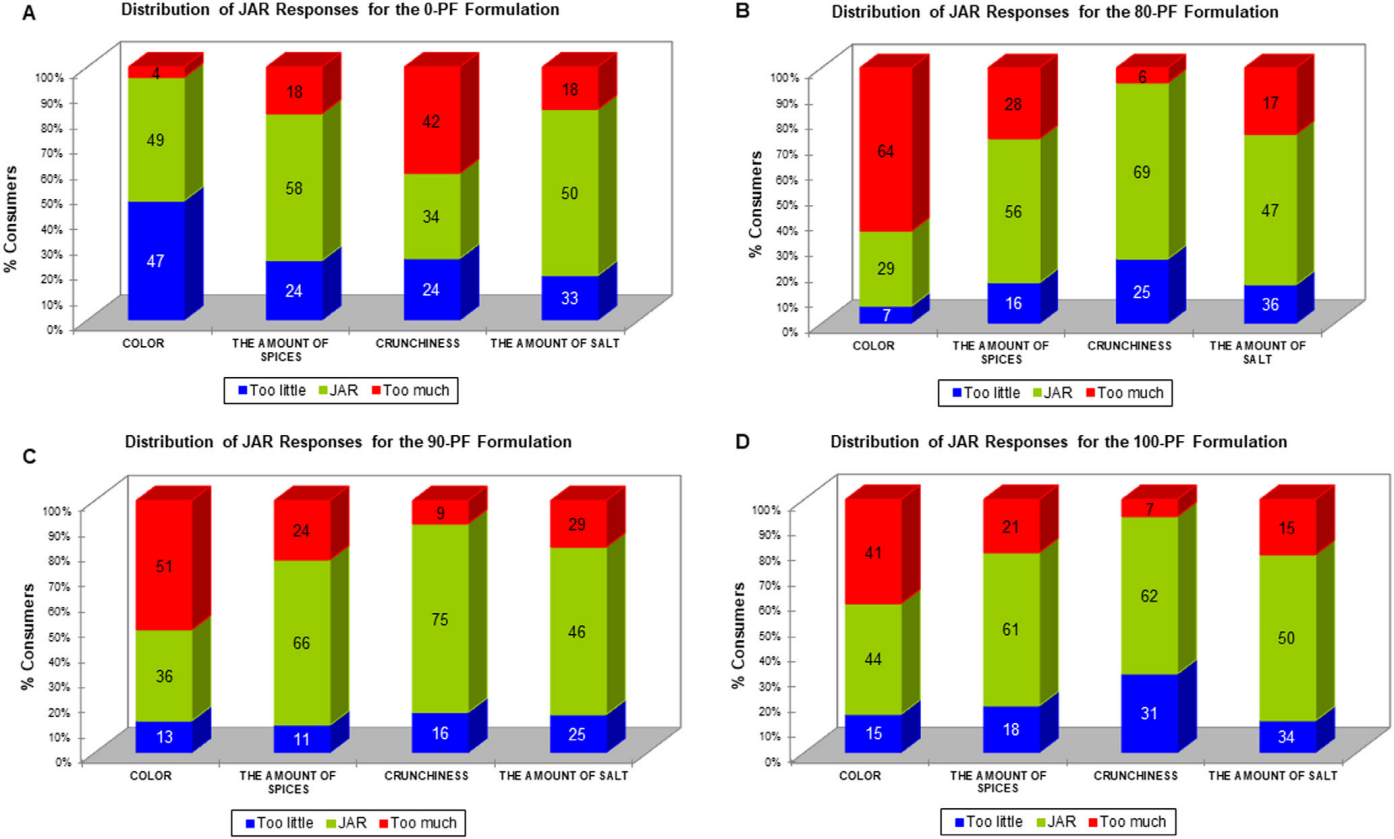
Frequency graph of sensory attributes of savory biscuit formulations assessment in just‐about‐right (JAR) methodology. (A) 0‐PF (control, 0% PF); (B) 80‐PF (formulation elaborated with MPF80); (C) 90‐PF (formulation elaborated with MPF90); and (D) 100‐PF (formulation elaborated with 100% PF). Perceived by more than 20% of consumers, with a significant decrease of at least 1 point in the average overall acceptability.

The data obtained through the JAR methodology were analyzed by evaluating the frequency of responses for six sensory attributes (crunchiness, color, spices and saltiness) using a five‐point scale. Significant differences (*P* < 0.05) were observed in the color attribute among the biscuit samples. As shown in the frequency distribution graph (Fig. 3), 64%, 51% and 41% of panelists indicated that the ‘color’ was darker than ideal for the 80‐PF, 90‐PF and 100‐PF formulations, respectively. In contrast, most assessors rated crunchiness as ideal, particularly in the 90‐PF and 100‐PF samples, which received ideal ratings exceeding 69%.

These findings are consistent with the results from the CATA analysis and overall acceptance scores, indicating that the texture and consistency of the enriched formulations were perceived as pleasant. By contrast, the 0‐PF formulation received a higher proportion of ratings above the ideal for crunchiness and texture attributes, with frequencies of 42% and 54%, respectively, resulting in a significant penalty score of 1.182 (*P* < 0.0001). Regarding spice and salt content, over 56% of participants rated these attributes as close to ideal across all samples, suggesting that the formulations displayed desirable sensory characteristics in these aspects.

## CONCLUSIONS

The present study demonstrates that green banana pulp flour and mixed pulp/peel flours can be effectively used as functional ingredients in savory biscuits, partially replacing sweet and sour cassava starches without compromising product quality. The enriched formulations showed clear improvements in nutritional composition, particularly in terms of dietary fiber and resistant starch content, compared to the control formulation. Structural analysis by 3D tomography revealed a denser and less porous matrix, especially in biscuits formulated with mixed pulp/peel flours at a 90:10 (pulp/peel) ratio, which was associated with improved textural properties and higher sensory acceptance. Overall, the results indicate that the combined use of green banana pulp and peel flours represents a viable and sustainable strategy to enhance the nutritional, structural, and sensory quality of savory biscuits, supporting their application in the development of functional baked products.

## CONFLICTS OF INTEREST

The authors declare that they have no conflicts of interest.

## Supporting information


**Table S1.** Absolute frequencies of sensory attributes checked for the different savory biscuit formulations, CATA terms (*n* = 120).
**Figure S1.** Effects on the color parameters of savory biscuits prepared with green banana pulp flour and pulp/peel mixed flours. 0‐PF (control, 0% PF); 80‐PF (formulation elaborated with MPF80); 90‐PF (formulation elaborated with MPF90) and 100‐PF (formulation elaborated with 100% PF).

## Data Availability

The data that support the findings of this study are available from the corresponding author upon reasonable request.
