# Supplementary material for: Savory biscuits formulated with mixed green banana pulp and peel flours: A sustainable approach to enhance nutritional, technological and sensory properties
Source: J Sci Food Agric. 2026 Mar 12;106(8):4951–61. doi: 10.1002/jsfa.70573 (PMC13157236; doi:10.1002/jsfa.70573)
Supplement: Supplementary file 1 — Table S1. Absolute frequencies of sensory attributes checked for the different savory biscuit formulations, CATA terms (n = 120). Figure S1. Effects on the color parameters of savory biscuits prepared with green banana pulp flour and pulp/peel mixed flours. 0‐PF (control, 0% PF); 80‐PF (formulation elaborated with MPF80); 90‐PF (formulation elaborated with MPF90) and 100‐PF (formulation elaborated with 100% PF). [file JSFA-106-4951-s001.docx]

**Supplementary material**

**Table S1** Absolute frequencies of sensory attributes checked for the different savory biscuit formulations, CATA terms (n = 120).

| Attributes | Samples | P-value from Cochran’s Q test | Average citation frequency (%) | Mean impact on acceptance |
| --- | --- | --- | --- | --- |
| Smooth taste ^(+)^ | 0-PF | 0.056 | 35 | 0.446 |
|  | 80-PF | 0.716 | 33 | 0.090 |
|  | 90-PF | 0.096 | 34 | 0.405 |
|  | 100-PF | 0.375 | 34 | 0.247 |
| Slightly salty taste ^(+)^ | 0-PF | **<0.001** | 45 | 0.951 |
|  | 80-PF | 0.356 | 47 | 0.214 |
|  | 90-PF | 0.566 | 48 | 0.131 |
|  | 100-PF | 0.168 | 50 | 0.364 |
| White to beige color ^(-)^ | 0-PF | 0.576 | 14 | 0.176 |
|  | 80-PF | 0.628 | 8 | 0.212 |
|  | 90-PF | 0.654 | 4 | 0.254 |
|  | 100-PF | 0.018 | 8 | 1.131 |
| Flavor of herbs or peppers ^(+)^ | 0-PF | **0.014** | 48 | 0.554 |
|  | 80-PF | **0.018** | 66 | 0.584 |
|  | 90-PF | **0.021** | 71 | 0.586 |
|  | 100-PF | 0.101 | 55 | 0.436 |
| Intense flavor of spices ^(-)^ | 0-PF | 0.953 | 19 | 0.016 |
|  | 80-PF | 0.629 | 25 | 0.128 |
|  | 90-PF | 0.812 | 29 | 0.060 |
|  | 100-PF | 0.182 | 25 | 0.405 |
| Bacon flavor  ^(+)^ | 0-PF | 0.909 | 6 | 0.054 |
|  | 80-PF | 0.479 | 8 | 0.295 |
|  | 90-PF | 0.518 | 8 | 0.266 |
|  | 100-PF | 0.738 | 7 | 0.176 |
| Herbal aroma ^(+)^ | 0-PF | 0.039 | 14 | 0.657 |
|  | 80-PF | 0.115 | 46 | 0.368 |
|  | 90-PF | 0.091 | 29 | 0.426 |
|  | 100-PF | 0.059 | 25 | 0.576 |
| Compact texture  ^(-)^ | 0-PF | **0.014** | 66 | 0.577 |
|  | 80-PF | 0.382 | 39 | 0.207 |
|  | 90-PF | 0.099 | 35 | 0.396 |
|  | 100-PF | 0.021 | 33 | 0.653 |
| Yellow to light orange color ^(+)^ | 0-PF | 0.828 | 45 | 0.048 |
|  | 80-PF | 0.585 | 9 | 0.218 |
|  | 90-PF | 0.434 | 8 | 0.337 |
|  | 100-PF | 0.623 | 13 | 0.189 |
| Yellow-gold color with brown dots ^(+)^ | 0-PF | 0.769 | 58 | 0.066 |
|  | 80-PF | 0.806 | 61 | 0.058 |
|  | 90-PF | 0.451 | 55 | 0.173 |
|  | 100-PF | 0.188 | 60 | 0.354 |
| Tasty biscuits and a good “snack” ^(+)^ | 0-PF | **<0.001** | 52 | 1.803 |
|  | 80-PF | **<0.001** | 48 | 1.139 |
|  | 90-PF | **<0.001** | 60 | 1.198 |
|  | 100-PF | **<0.001** | 47 | 1.220 |
| Light biscuits ^(-)^ | 0-PF | 0.810 | 34 | 0.056 |
|  | 80-PF | 0.586 | 39 | 0.129 |
|  | 90-PF | 0.248 | 51 | 0.265 |
|  | 100-PF | 0.981 | 45 | 0.006 |
| Very salty taste ^(-)^ | 0-PF | 0.002 | 11 | 1.115 |
|  | 80-PF | 0.678 | 14 | 0.137 |
|  | 90-PF | 0.000 | 15 | 1.284 |
|  | 100-PF | 0.994 | 10 | 0.003 |
| Pleasant and addictive taste ^(+)^ | 0-PF | **<0.001** | 30 | 2.086 |
|  | 80-PF | **<0.001** | 33 | 1.234 |
|  | 90-PF | **<0.001** | 39 | 1.317 |
|  | 100-PF | **<0.001** | 34 | 1.301 |
| Slim and “flat” shape ^(+)^ | 0-PF | 0.166 | 73 | 0.346 |
|  | 80-PF | 0.119 | 66 | 0.383 |
|  | 90-PF | 0.309 | 67 | 0.248 |
|  | 100-PF | 0.494 | 65 | 0.188 |
| Mild taste of spices ^(+)^ | 0-PF | 0.039 | 35 | 0.483 |
|  | 80-PF | 0.489 | 31 | 0.173 |
|  | 90-PF | 0.097 | 42 | 0.386 |
|  | 100-PF | 0.058 | 39 | 0.515 |
| Cheese flavor ^(+)^ | 0-PF | 0.005 | 61 | 0.652 |
|  | 80-PF | 0.000 | 25 | 1.075 |
|  | 90-PF | 0.067 | 22 | 0.509 |
|  | 100-PF | 0.175 | 23 | 0.428 |
| Baked biscuit aroma ^(+)^ | 0-PF | 0.006 | 46 | 0.615 |
|  | 80-PF | 0.000 | 25 | 1.075 |
|  | 90-PF | 0.253 | 36 | 0.272 |
|  | 100-PF | 0.139 | 37 | 0.404 |
| Toasted bread aroma ^(+)^ | 0-PF | 0.770 | 17 | 0.086 |
|  | 80-PF | 0.408 | 40 | 0.195 |
|  | 90-PF | 0.937 | 33 | 0.019 |
|  | 100-PF | 0.417 | 26 | 0.243 |
| Crunchy texture ^(+)^ | 0-PF | **0.014** | 50 | 0.548 |
|  | 80-PF | 0.152 | 23 | 0.397 |
|  | 90-PF | 0.103 | 82 | 0.482 |
|  | 100-PF | 0.207 | 70 | 0.362 |

*Positive(+) and negative (–) signs indicate the direction of the attribute’s influence on acceptance. Significance level indicated by *** if p < 0.001, ** if p < 0.01 and * if p < 0.05. 0-PF (control, 0% PF); 80 – PF (formulation elaborated with MPF80); 90 – PF (formulation elaborated with MPF90) and 100 – PF (formulation elaborated with 100% PF).


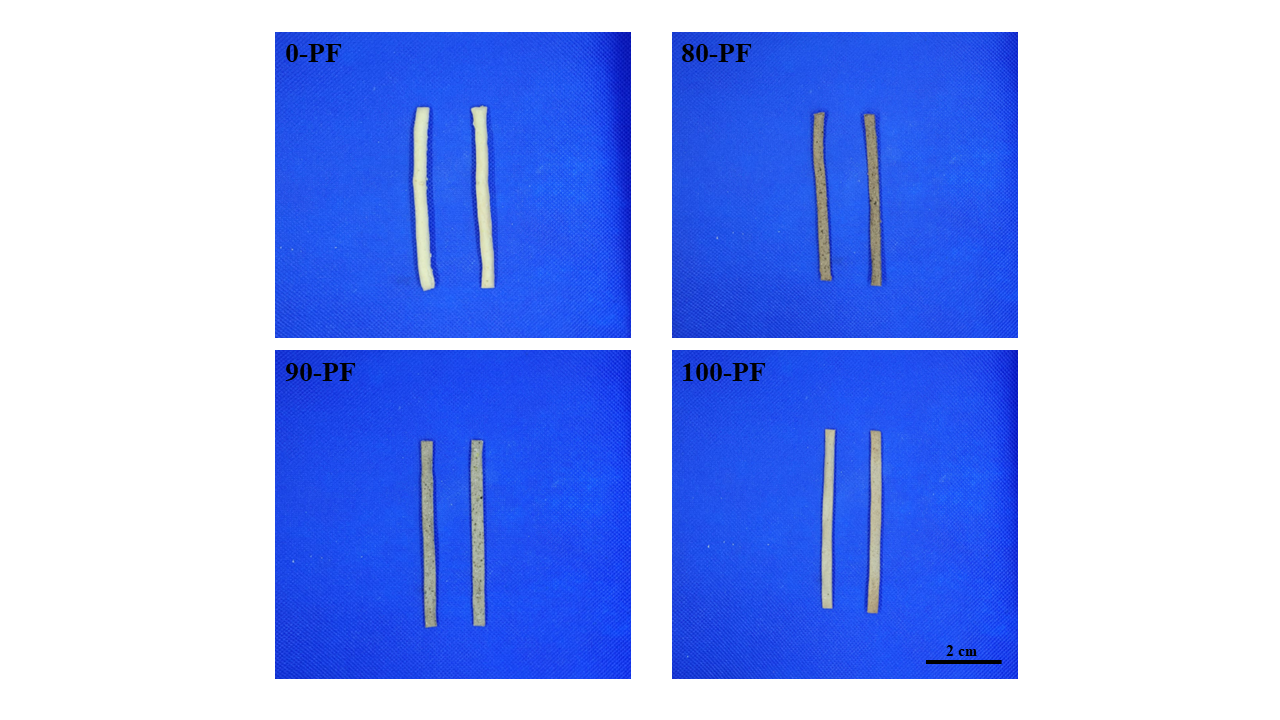


**Figure S1** Effects on the color parameters of savory biscuits prepared with green banana pulp flour and pulp/peel mixed flours. 0-PF (control, 0% PF); 80 – PF (formulation elaborated with MPF80); 90 – PF (formulation elaborated with MPF90) and 100 – PF (formulation elaborated with 100% PF).
